# Insights into the 3D permeable pore structure within novel monodisperse mesoporous silica nanoparticles by cryogenic electron tomography†

**DOI:** 10.1039/d3na00145h

**Published:** 2023-04-18

**Authors:** Yidong Xia, Jianfang Liu, Rahul Kancharla, Jiaoyan Li, Seyed M. Hatamlee, Gang Ren, Viktoriya Semeykina, Ahmed Hamed, Joshua J. Kane

**Affiliations:** a Energy and Environment Science & Technology, Idaho National Laboratory Idaho Falls 83415 ID USA yidong.xia@inl.gov +01 208 526 7490; b Molecular Foundry, Lawrence Berkeley National Laboratory Berkeley 94720 CA USA; c Materials & Fuels Complex, Idaho National Laboratory Idaho Falls 83415 ID USA; d Department of Mechanical and Aerospace Engineering, University at Buffalo Buffalo 14260 NY USA; e Department of Chemistry, The University of Utah Salt Lake City 84112 UT USA

## Abstract

Sintered agglomerate of synthetic mesoporous silica nanoparticles (MSNs) is an architected geomaterial that provides confinement-mediated flow and transport properties of fluids needed for environmental research such as geological subsurface energy storage or carbon capture. The design of those properties can be guided by numerical simulations but is hindered by the lack of method to characterize the permeable pores within MSNs due to pore size. This work uses the advances of an Individual Particle cryogenic transmission Electron Tomography (IPET) technique to obtain detailed 3D morphology of monodispersed MSNs with diameters below 50 nm. The 3D reconstructed density-maps show the diameters of those MSNs vary from 35–46 nm, containing connected intraparticle pores in diameter of 2–20 nm with a mean of 9.2 ± 3 nm, which is comparable to the mean interparticle pore diameters in sintered agglomerate. The characterization of the pore shape and dimensions provides key information for estimating the flow and transport properties of fluids within the sintered agglomerate of those MSNs and for modeling the atomic MSN structures needed for pore-fluid simulations.

## Abstract 2

Synthetic mesoporous silica nanoparticle (MSN) is an architected geomaterial that provides confinement-mediated flow and transport properties of fluids needed for environmental research such as subsurface energy storage or carbon capture.

## Graphical abstract

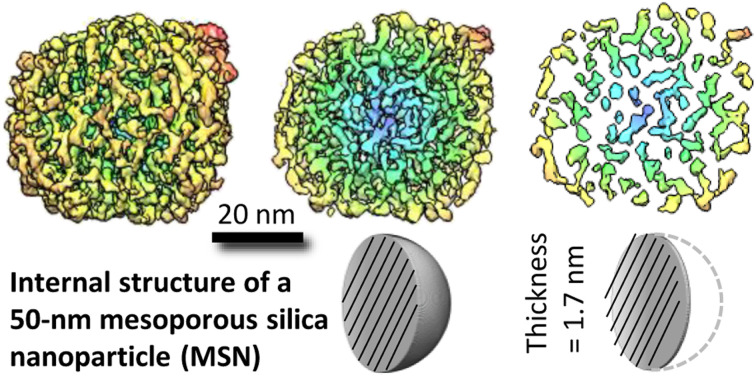

## 1. Introduction

Flow experiments^1^ and molecular dynamics (MD) simulations^2^ demonstrated that water flows in a carbon nanotube much faster than computational fluid dynamics model predictions.^3^ These discoveries drew attention to the nanoconfinement effect on fluid properties, which can be harnessed for applications such as energy source recovery from nanoporous tight shale,^4^ water desalination,^5–7^ and drug delivery.^8^ The needs for understanding flow and transport in nanoporous geomaterials are further driven by carbon reduction applications such as subsurface storage of CO_2_. For flow studies, however, the use of natural nanoporous geomaterials entails challenges. Pore analysis of shale samples based on focused ion beam (FIB) scanning electron microscopy (SEM) nano-tomography^9^ shows that the spatial distributions of the pores are highly heterogeneous, ranging from tens to hundreds of nanometers in irregular sizes and shapes. Flow simulations using heterogeneous pore networks reconstructed from FIB-SEM imaging of shale samples must choose regions of interest (ROI), where pore size cannot be controlled and subsequently the results may not be reproduced from one ROI to another.^10,11^ Instead, synthetic nanoporous geomaterials with tunable pore size and distributions are preferred.

Amorphous (non-crystalline) silica has gained attention in subsurface sciences for its use in constructing tunable nanoporous geomaterial. Porous solids are classified as mesoporous when pore size ranges from 2–50 nm.^12–14^ With increasing use of nanopore and nanoporous in literature, we use these terms interchangeably with mesopore and mesoporous here. Synthetic mesoporous silica particles such as commercially available MCM-41 (ref. 15 and 16) and SBA-15 (ref. 17 and 18) are widely used to construct controlled nanoporous geomaterials *via* sintering, for studying properties of nanoconfinement fluids. For example, SBA-15, with its pore size varied normally from 4–12 nm, is extensively studied due to its high surface area, thick framework walls and straight cylindrical pores.^19^ Those materials have been used for investigating structural and transport properties of fluids in ordered mesopores with approximate or idealized pore shapes, *via in situ* neutron scattering measurements and MD simulations.^20–24^ Interests are also drawn to probing the mechanistic understandings of synthesis and thermal-chemical induced evolution of silica mesopore morphology.^25–27^ However, SBA-15 has undesired features for flow experiments. For example, the size of ordered pores inside the particles is often not ideal and can have disordered regions and defects. Also, the particle size is non-uniform and can vary from a few hundred nanometers to tens of microns. Recent advances in 3D printing to fabricate SBA-15 (ref. 28) may present a future solution to those quality concerns. Currently, sintering these particles can result in highly non-uniform permeability lacking control of pore size or distribution. Though those issues will normally not affect applications such as adsorption or catalysis, they must be avoided for reproducible flow experiments, for which the ideal particles are expected to have connected intraparticle mesopores with diameters in single-digit nanometers and interparticle pores of similar size in sintered agglomerate.

**Fig. 1:**
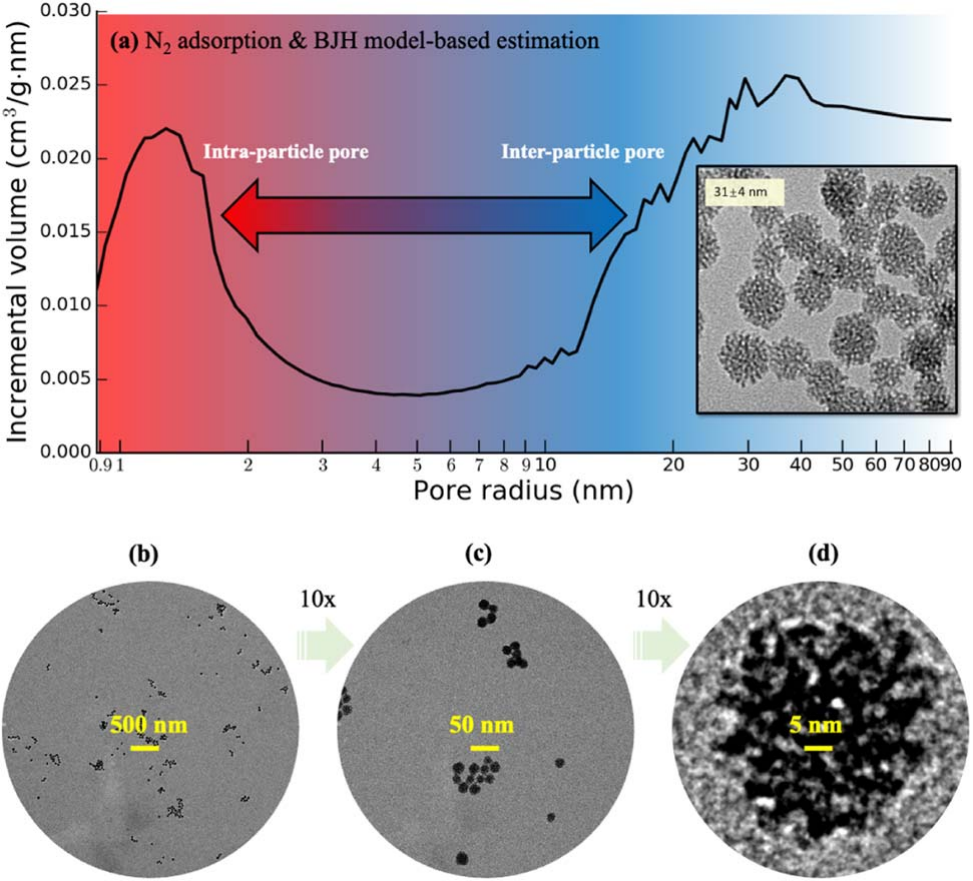
(a) Pore size estimate from a pack of mesoporous silica nanoparticles based on the experimental N_2_ adsorption measurement and BET/BJH models. (b)–(d) TEM images of air-dry sample of dispersed mesoporous silica nanoparticles.

Recent progress was made in the synthesis of mesoporous silica nanoparticles (“MSNs”) with controllable particle size, pore size, and pore volume.^29^ Those MSNs are nearly monodisperse and have a tunable diameter as small as 30 nm, which are suitable for flow studies regarding the possibility of minimizing size difference between intraparticle and interparticle pores in sintering. Fig. 1 shows the transmission electron microscope (TEM) images of those MSNs with diameters measured in 31 ± 4 nm. However, the existing mesoscopic flow models assumed idealized or simplified particle surfaces of these MSNs for pore-flow simulations,^30,31^ since 3D imaging approaches based on nano-CT^31^ or FIB-SEM^10,11^ cannot provide sufficient resolution. This work reports the 3D morphology of those monodisperse MSNs using an individual particle cryogenic electron tomography (IPET) technique^32–34^ with missing-wedge correction.^35^ Electron tomography for materials science has a significant advancement over the recent decades,^36^ including atomic resolution STEM tomography of nanoparticles.^37^ Various sample preparation techniques have been explored to improve the efficiency of electron tomography, such as the approach by Padgett *et al.*,^38^ in which nanofibers are used to collect a 180° tilt series of nanoparticles. In this study, we apply the conventional cryo-ET technique used in biology to study the MSN samples. To avoid potential structural damage of the mesopores within the MSNs, the MSN samples are frozen in the vitreous ice. This also enables higher throughput, allowing tilt series to be collected from more particles in a given timeframe. Our method provides sufficient capability to capture information on pore size and distribution, which are the target resolutions. Although the 3D scanning TEM imaging-based characterization of a commercially available 2 μm (2000 nm) sized mesoporous silica sphere was recently reported,^39^ this work is the first-of-its-kind that presents characterization of monodisperse MSNs with size below 50 nm. The extremely small sizes of these MSNs require a novel and more rigorous sample preparation method for TEM, which will be introduced in this work. Moreover, this work presents a substantially simplified image processing technique for MSNs that eliminates the need for an artifact-prone multi-step image correction procedure involving background correction, charge correction and linearity correction.^39^ In addition, this work presents the detailed 3D mesopore visualization within the MSNs for the first time. Most remarkably, the obtained 3D imaging data of these MSNs enables the construction of detailed MSN structures urgently needed not only by flow studies but also by thermodynamics,^40^ reactivity,^41,42^ and geomechanics^43,44^ studies of nanoconfined fluids, serving the scientific value way beyond the nanoscale MSN imaging and characterization technique itself.

## 2. Materials and methods

### 2.1 Nanoparticle synthesis

MSN colloids are prepared with the following procedures.^29^ First, 10 ml of deionized water is mixed with triethanolamine (TEA) and cetyltrimethylammonium bromide (CTAB) and equilibrated at 60 °C. Then, 1.5 ml of tetraethylorthosilicate (TEOS) is added to the mixture under vigorous stirring, and the reaction is left to stir at 60 °C for 24 hours. The typical reagent molar ratio is Si : CTAB : TEA : H_2_O = 1 : 0.06 : 0.02–0.08 : 80. The particles grow predominantly through the aggregation of silica clusters and their final sizes are mainly determined by the moment when they reach electrostatic or steric stabilization. The obtained MSNs are washed with acidic ethanol solution of 0.01 M HCl six times using centrifugation–redispersion cycles to remove surfactant from the mesopores. The shapes of MSNs are examined using a JEOL JEM-1400 TEM with an acceleration voltage of 120 kV. The average particle diameter are calculated by measuring 150 to 250 particles using ImageJ.^45^ Specific surface area and pore size distribution are roughly estimated based on the Brunauer–Emmett–Teller (BET) model^46,47^ and Barrett–Joyner–Halenda (BJH)^48,49^ model, respectively, with source data from low-temperature N_2_ adsorption measurements using a Micromeritics ASAP 2020 Adsorption Analyzer. Finally, a degassing procedure is carried out at 200 °C for 4 hours under 1 μm Hg vacuum, with the isotherm recorded in the pressure range of *P*/*P*_0_ = 0.002–0.99, where *P*_0_ is the saturation pressure at which N_2_ starts capillary condensation in the pores at a given temperature. The resultant MSNs exhibit a narrow particle diameter distribution (31 ± 4 nm) and polydispersity index (∼0.13) based on 2D TEM images, as well as well-developed mesopores.^29^ The N_2_ adsorption measurement data shows that the intraparticle pore size, defined as radius of the liquid N_2_ meniscus, ranges roughly in 1–2 nm based on the cylindrical pore approximation in the BJH model, shown in Fig. 1a. Following this method, the total pore volume of a pack of dried MSNs is estimated to exceed 1 cm^3^ g^−1^, in which the intraparticle pore volume accounts for about 40% and the rest is interparticle pore volume with a minimal size above 5 nm estimated from the wide downward peak from 5 to 20 nm in Fig. 1a. Fig. 1a is only a rough estimate of pore sizes, as the size and volume of interparticle pores depend on packing, and the cylindrical pore assumption is not suitable for the present MSNs.

### 2.2 TEM sample preparation and data acquisition

The 3D imaging of MSNs with detailed internal porous structure is made possible with cryogenic electron microscopy (cryo-EM). Though the general procedure of cryo-EM 3D imaging was established in previous works for other materials,^33,34^ this is the first time that it was adapted for MSNs. Thus, it is necessary to briefly describe the major steps with specific apparatus and processing parameters determined as follows. First, the cryo-EM grids and air-dry EM grids were prepared, respectively, by the dissolved MSN samples in a concentration of 2 mg ml^−1^, in which 1 mg powder sample was added into 500 μl ammonia solution followed by 2 hours of ultrasonic. For cryo-EM grid preparation, an aliquot (4 μl) of sample solution was placed on a single layer graphene oxide support film on lacey carbon, 300 mesh copper grid (Ted Pella Inc., Redding, CA, USA) that was priorly glow-discharged for 15 s. After blotting with filter paper from one side for 3.5 s, the grid was flash-frozen in liquid ethane at ∼90% humidity and 4 °C with a Leica EM GP rapid-plunging device (Leica, Buffalo Grove, IL, USA) before transferred into liquid nitrogen for storage. For air-dry EM grid preparation, an aliquot (4 μl) of sample solution was placed on an ultra-thin carbon film grid (CF-200-Cu-UL, Electron Microscopy Sciences) that was priorly glow-discharged for 15 s. After 1 minute incubation, excess solution on the grid was removed by filter paper. The grid was then air-dried with nitrogen. The cryo-EM specimens were imaged by a Titan Krios G3i TEM (ThermoFisher Scientific) with a Gatan energy filter (Gatan, Inc., Pleasanton, CA, USA), operated under 300 keV. Micrographs were acquired on a Gatan K3 direct electron detector operated in correlated double sampling (CDS) mode and super-resolution mode at a nominal magnification of 53k× (*i.e.*, ∼1.67 Å per pixel) with a defocus of ∼9 μm by SerialEM.^50^ The tilt series were acquired from −60° to +60° in a tilting step of 2° and total dose of ∼888 e^−^ Å^−2^ (at exposure time of ∼5.0 s per tilt image). The exposures are proportional to the inverse cosine of the tilt angle to the half power, in which the dose at 60° was 1.41 times of dose at 0°. The vitreous ice was found to be radiation damaged, while no observable radiation damage was detected on the MSNs. The air-dry EM grid was examined using a Zeiss Libra 120 Plus TEM (Carl Zeiss NTS, Overkochen, Germany), which was equipped with a LaB_6_ gun operating at 120 kV, an in-column energy filter, and a 4k × 4k Gatan UltraScan 4000 CCD camera.

### 2.3 IPET 3D reconstruction

The multi-frame cryo-EM image at each tilt-angle was motion corrected by MotionCor2.^51^ The tilt series of the whole micrographs were initially aligned using IMOD.^52^ The Contrast Transfer Function (CTF) was determined by GCTF^53^ and corrected by TOMOCTF.^54^ To reduce image noise, tilt series were further conducted using a median-filter software package and a contrast enhancement method.^55^ The method involves a multi-scale image decomposition algorithm based on edge-preserving smoothing, which effectively identifies objects in the presence of high levels of noise. Moreover, this method enhances the contrast of object images without introducing additional noise or causing a significant decrease in image resolution. Each targeted MSN was boxed from the above whole-micrograph tilt series to a small-size tilt series (192 × 192 pixel, 3.34 Å per pixel) for IPET 3D reconstruction.^56^ Briefly, an *ab initio* 3D map was generated as an initial model by back-projecting the small-size tilt series. During the iteration and refinement processes, a set of Gaussian low-pass filters, soft-boundary circular and particle-shaped masks were automatically generated and sequentially applied to the tilt series and projections of the references. To reduce missing-wedge artifact caused by the limited tilt angle range, the 3D maps were processed by a low-tilt tomographic 3D reconstruction method (LoTToR).^35^ Finally, all IPET 3D reconstructions were low-pass filtered to 15 Å and rendered in UCSF Chimera.^57^ The resolution of the refined 3D model was estimated by Fourier shell correlation (FSC) between two 3D density maps reconstructed independently from odd and even number tilt-angle series. The resolution based on (a) the 0.143 threshold or (b) the 0.5 threshold is reported.

## 3. Results and discussion

### 3.1 3D structure of MSNs

**Fig. 2:**
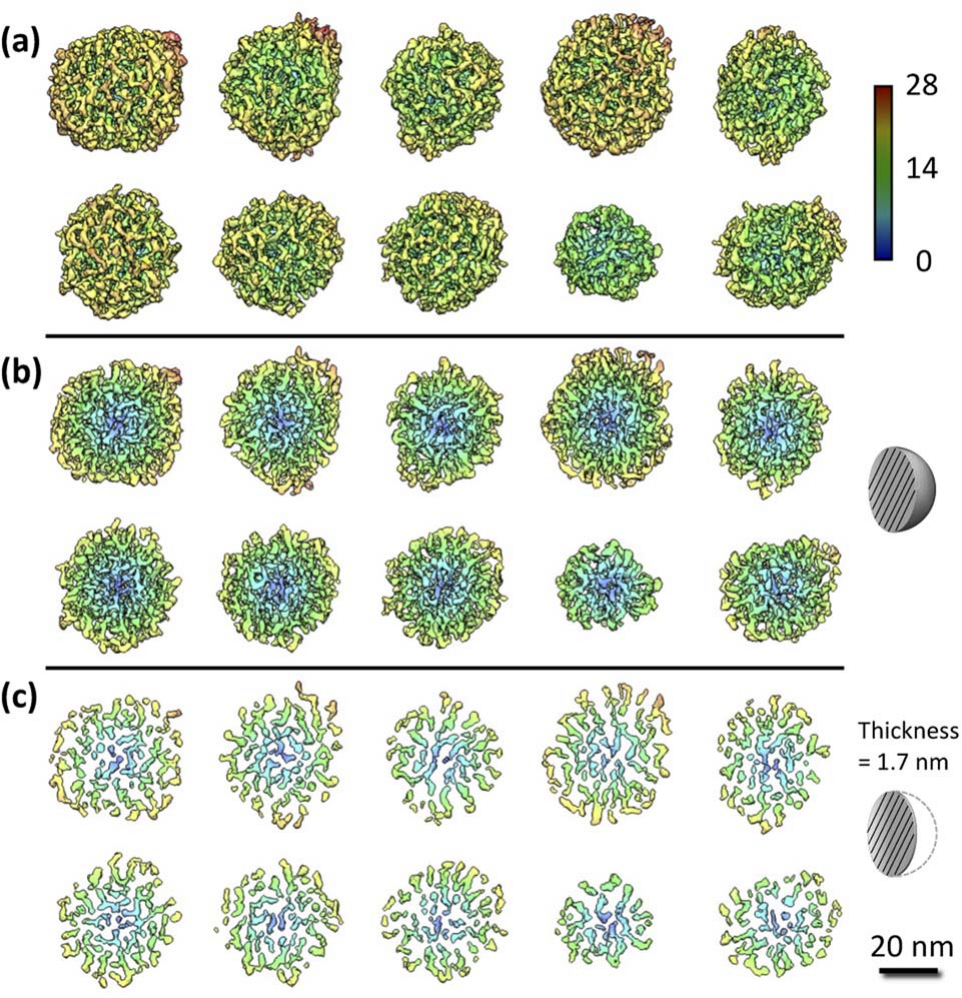
IPET results of the MSN samples: (a) 3D maps of individual MSNs where solids are colored using distance from particle geometric centers [Å]; (b) cross-section view; (c) slice view. Additional details of the process to obtain these graphs are provided in ESI A.†

Fig. 2a shows ten rendered 3D density maps of MSNs in surface color by radius from inner (blue) to outer (red). The diameters of those MSNs are in 35–46 nm and larger than those measured from 2D TEM images (31 ± 4 nm).^29^ The cross-section views in Fig. 2b show the pore structure throughout the interior of those MSNs. Fig. 2c shows extract slices in the middle of those MSNs with thickness of 1.7 nm. For clarity, we take the first MSN (top left corner in Fig. 2a) as an example to describe the use of the procedure in the previous section to obtain the 3D map. First, the tilt images of the MSN were aligned to a global center iteratively to achieve a final *ab initio* 3D reconstruction. The representative tilt images are shown in ESI A (Fig. S1†). A step-by-step refinement was then conducted. After low-pass filtering to 15 Å, the final 3D density map was constructed with the resolution estimated as 18 Å based on FSC and a criterion of 0.143. The resultant overall dimension of the MSN is ∼44 nm. By repeating the above process, another nine MSNs were 3D reconstructed with the resolution in 18–20 Å. Additional details are provided in ESI A (Fig. S2–S6†).

### 3.2 Porosity of MSNs

**Fig. 3:**
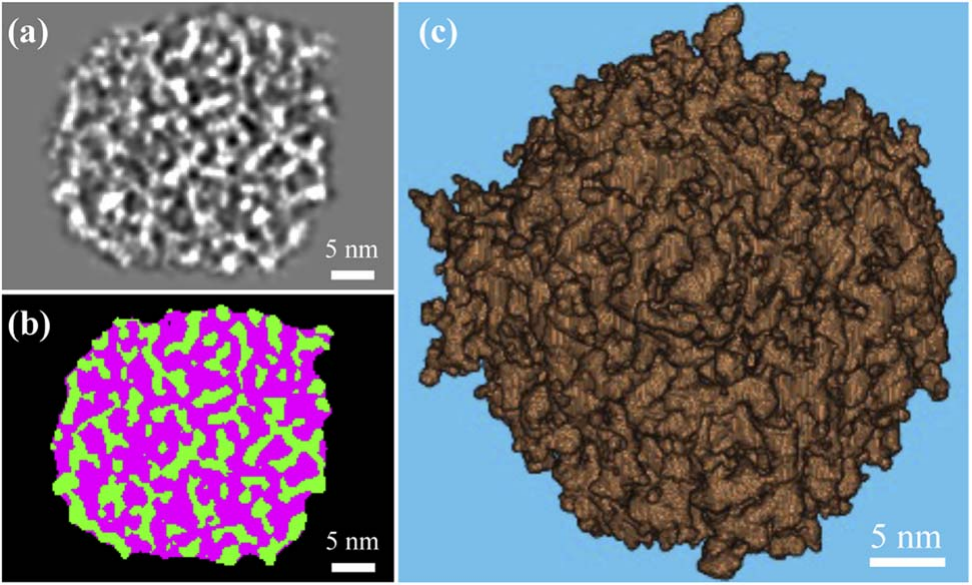
Display of an axial cross-section slice showing one mesoporous silica nanoparticle approximately from the middle of the volume: (a) grayscale graph before segmentation and (b) binarized graph after segmentation and morphological processing showing silica solid in the green color and pore structure in the pink color. (c) 3D rendering of the solid silica after segmentation.

The *k*-means clustering algorithm^58^ was used to perform segmentation on each 16 bit grayscale image stack based on intensity difference observed between solid silica and background (*i.e.*, pore structure and volume external to the particle). A 3D array mapped from each image stack was partitioned into five clusters based on grayscale values. By keeping the two clusters with the largest mean grayscale values, the solid silica was segmented from the background. To isolate intraparticle pore structures from solid, we performed a morphological closing process with a 7 × 7 × 7 voxel mask (determined in a sensitivity study by incrementally growing the mask edge size from 3 pixels, until the resultant porosity did not vary over 5% from the previous one) and then a morphological filling process to remove any isolated solid within the pores. A middle cross-section slice of a MSN in grayscale and after segmentation is shown in Fig. 3a and b, respectively. In Fig. 3b, the green color and pink colors, respectively, represent the solid and pore volume. Fig. 3c shows the 3D rendering of solid phase after segmentation. Based on the segmented 3D particle volume of individual MSNs, the average intraparticle porosity is 57% with 95% confidence.

### 3.3 Mesopore size and tortuosity of MSNs

**Fig. 4:**
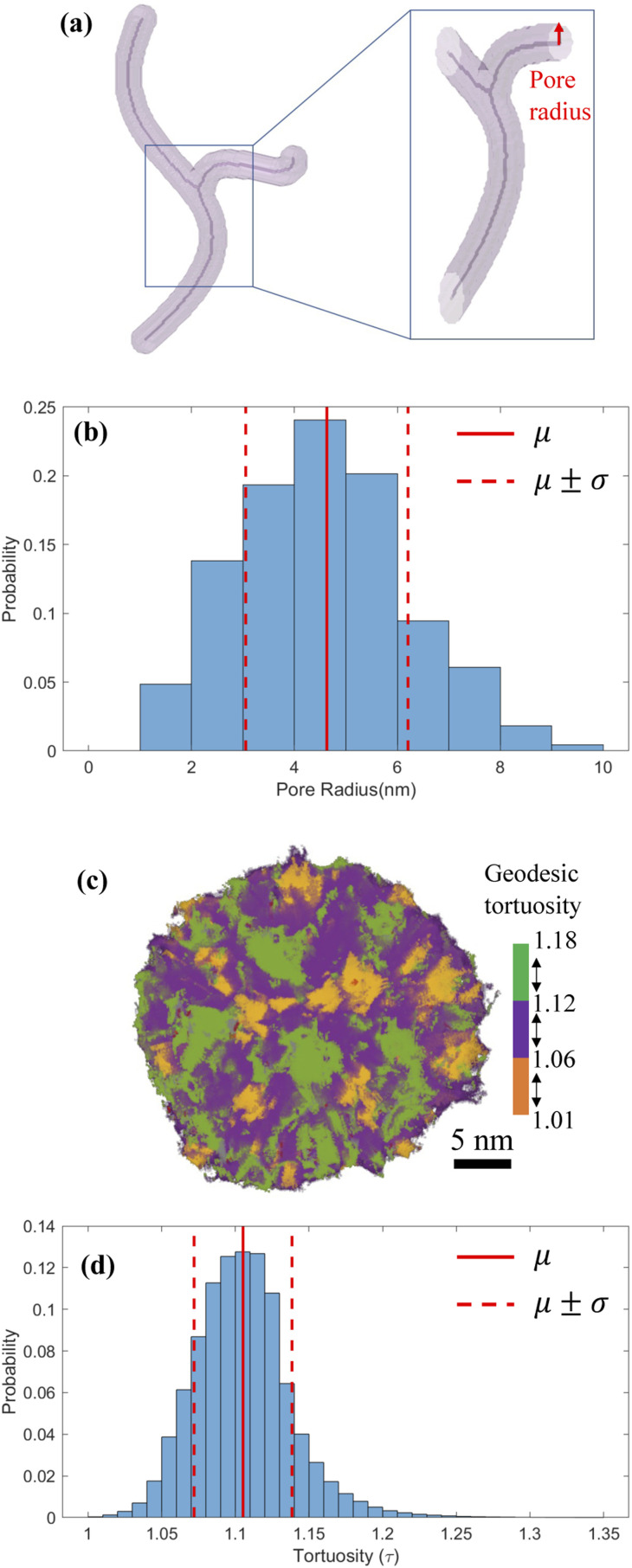
(a) An example of synthetic pore volume showing pore skeleton, with a zoom-in graph showing how pore radius from the skeleton is estimated. (b) Distribution of calculated intraparticle pore radius in the reconstructed MSNs. The solid red line indicates the mean value *μ* and two dashed red lines from *μ* − *σ* to *μ* + *σ* encompass the standard deviations. (c) An example of visualization of geodesic tortuosity of an MSN, where the orange color represents the tortuosity value in [1.01, 1.06), the purple color in [1.06, 1.12), and the green color in [1.12, 1.18). (d) Distribution of geodesic tortuosity of intraparticle pore volume in MSNs. The solid red line indicates the mean value *μ* and two dashed red lines from *μ* − *σ* to *μ* + *σ* encompass the standard deviations.

Based on the images of segmented 3D particle volume, we used skeletons to characterize the morphology and size distribution of intraparticle pores.^59,60^ Skeletons provide a medial axis representation for a shape-preserving volume, which retains the morphology of the original volume. Fig. 4a shows a synthetic pore volume with its skeleton visualized along the medial axis. After the skeleton is extracted, local pore radii are calculated by estimating the Euclidean distance from a point on the skeleton to pore surface, shown by a red arrow in Fig. 4a. Fig. 4b shows the radius of connected pores inside the reconstructed MSNs varies in 1–10 nm with a mean of 4.6 ± 1.5 nm, which is larger than the rough estimates in Fig. 1a but within the 2–30 nm mesopore size range of original surfactant micelles.^29^ Simulations of pressure sintering of idealized 30 nm-diameter solid spheres based on the discrete element method^61–63^ show the mean interparticle pore radius of 4.3 ± 0.7 nm.^30^ It is close to the mean of the present MSN intraparticle pore radius (4.6 ± 1.5 mm), indicating that the permeable pathways within these MSNs should not be ignored when estimating the flow and transport properties of fluids in sintered agglomerate of these MSNs. Previous studies used to neglect the intraparticle porosity of MSNs, because of either insufficient resolution or its negligible impact on large MSNs. For example, sintering of 100 nm-diameter MSNs would result in interparticle pores with diameters in 25–30 mm, for which the nanoconfinement effect on fluid flow is weak.^64^ The geodesic tortuosity (GT) of the MSNs is calculated to describe the relative fluid travel distance through a pore network compared to the nominal particle dimensions. GT is the ratio of geodesic distance (the shortest path of two points constrained inside pores)^65^ and Euclidean distance (the straight-line distance between two points).^66^ For example, Fig. 4c depicts the paths in one MSN with three colors to represent the segmented GT ranges. Fig. 4d shows GT within the MSN samples is 1.11 ± 0.03. The low value and narrow distribution in GT indicate homogeneous flow and transport inside these MSNs.

### 3.4 All-atom model

**Fig. 5:**
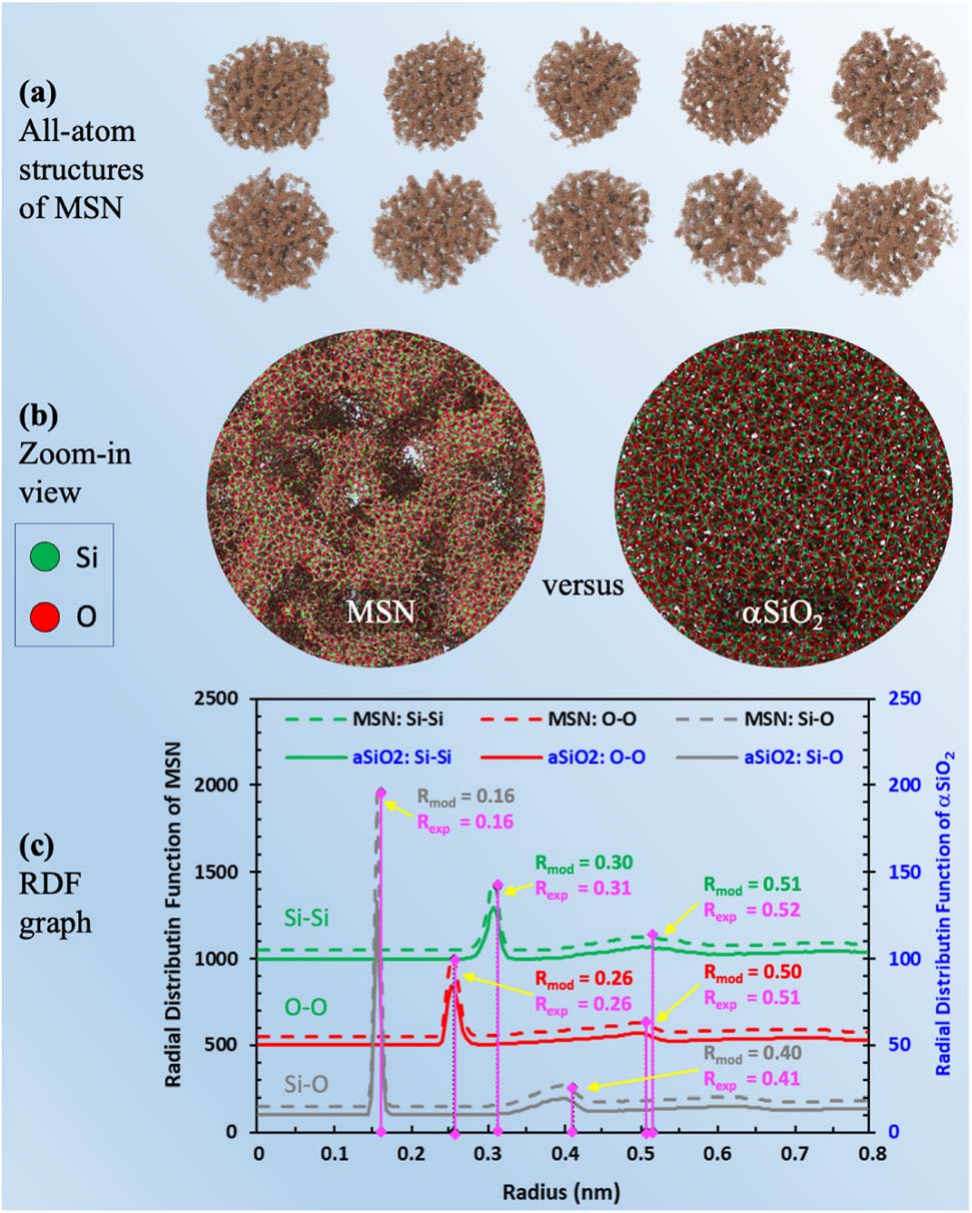
(a) Visualization of molecular simulation-derived all-atom structures of MSN based on the segmented 3D images of MSN; (b) comparison of zoom-in visualizations of the MSN mesopore structure and matrix material (aSiO_2_) with no pore structure; (c) plot of RDF for MSN and aSiO_2_ after isothermal equilibration. In each RDF profile, the critical radii (*R*_mod_) corresponding to the first two peaks match the experimental measurements (*R*_exp_).^69^ Note that the RDF values of the peaks are not critical. It is the radius of the peaks that informs the bond length of each pair.

The last and most important usage of 3D image data of MSNs is for providing access to atomic structures of MSNs to enable studies of the properties of fluids confined in MSNs. We present approximate all-atom models of the present MSNs based on segmented 3D images from IPET and MD simulations using LAMMPS^67^ as follows. MD simulations of a heating and annealing process were performed in a 50 nm cubic domain of crystalline β-quartz with periodic boundaries, where the Si–Si, Si–O, and O–O atomic interactions were described by the CLAYFF^68^ force field. The crystalline β-quartz was first equilibrated at 300 K for 1 ns using the canonical (NVT) ensemble. The system temperature was then increased to 5000 K (above the melting point of crystalline silica) in 1 ns and maintained for another 1 ns. Next, the system temperature was decreased to 300 K by 2 K per ps, maintained at 300 K for 1 ns using the isothermal–isobaric (NPT) ensemble, and for another 1 ns using the NVT ensemble to minimize the potential energy for reaching the stable amorphous phase of silica (“αSiO_2_”). The resultant domain of αSiO_2_ was superimposed to the segmented 3D image block of each MSN sample. Only the atoms of αSiO_2_ matrix residing in solid voxels (*e.g.*, green color in Fig. 3b) were kept. This procedure also resulted in some isolated atoms (*e.g.*, silicon atoms with 4 dangling bonds or oxygen atoms with 2 dangling bonds), which were then removed. The resultant all-atom MSN structures are shown in Fig. 5a. Zoom-in view inside an MSN in contrast to αSiO_2_ matrix is shown in Fig. 5b. The radial distribution functions (RDF) of Si–Si, Si–O, and O–O bonds in the all-atom MSN structures are shown in Fig. 5c and compared with the αSiO_2_ matrix. In each RDF profile, the first two peaks correspond to the critical radii. The critical radii of MSNs match those of αSiO_2_ matrix and are close to the experimental data of αSiO_2_ matrix,^69^ indicating that the obtained all-atom MSN structures are stable to use as morphological data (available in ESI B†) for various studies. Notice that the difference in the peak values of RDFs between the MSN and αSiO_2_ at the same locations of critical radius, *e.g.*, at 0.16 and 0.26 nm, is attributed to the different sample sizes of the MSN (about 50 nm in diameter) and αSiO_2_ (about 5 nm in diameter) in this comparison. The peak value of RDF is relatively irrelevant to the resultant bond lengths.

## 4. Conclusion

This work has used an IPET technique to obtain detailed 3D morphology of monodispersed MSNs with diameters below 50 nm. It provides an unprecedented visualization of MSN morphology and intrapore size estimation. Results show the diameters of those MSNs vary in 35–46 nm, while the diameters of connected intrapores in these MSNs vary in 2–20 nm with a mean of 9.2 ± 3 nm, which are close to the mean interparticle pore diameters (8.6 ± 1.4 nm) in sintered agglomerate. This suggests that both intraparticle and interparticle pores must be considered when estimating the flow and transport properties of fluids in sintered agglomerate of those MSNs. The atomic structure models of these MSNs obtained from the imaging data are invaluable inputs for various studies of properties of confined fluids.

## Data availability

A: Additional details of the process to obtain reconstructed 3D density maps of individual MSNs from IPET (ESI†). B: Individual all-atom MSN structure data files in the Protein Data Bank (pdb) file format (ESI†). C: Images of the raw tilt series available on Figshare https://doi.org/10.6084/m9.figshare.22403149.v1.

## Author contributions

Yidong Xia: funding acquisition, project administration, conceptualization, supervision, investigation, formal analysis, visualization, writing – original draft. Jianfang Liu: methodology, investigation, data curation, formal analysis, validation, visualization, writing – original draft. Rahul Kancharla: methodology, data curation, formal analysis, software, visualization, writing – original draft. Jiaoyan Li: supervision, formal analysis, writing – original draft. Seyed M. Hatamlee: investigation, data curation, visualization. Gang Ren: resources, supervision, methodology, writing – review & editing. Viktoriya Semeykina: resources, methodology, investigation, writing – original draft. Ahmed Hamed: writing – review & editing. Joshua J. Kane: conceptualization, resources, software, supervision, methodology, writing – review & editing.

## Conflicts of interest

There are no conflicts to declare.

## Supplementary Material

NA-005-D3NA00145H-s001

NA-005-D3NA00145H-s002
