# Supplementary material for: Insights into the 3D permeable pore structure within novel monodisperse mesoporous silica nanoparticles by cryogenic electron tomography
Source: Nanoscale Adv. 2023 Apr 18;5(11):2879–86. doi: 10.1039/d3na00145h (PMC10228490; doi:10.1039/d3na00145h)
Supplement: NA-005-D3NA00145H-s001 [file NA-005-D3NA00145H-s001.pdf]

Electronic Supplementary Material (ESI) for Nanoscale Advances.
This journal is © The Royal Society of Chemistry 2023

Supporting Information A for

Insights into the 3D connected pore structure within monodisperse mesoporous silica nanoparticles by cryogenic electron tomography

Yidong Xia,[*,†,#] Jianfang Liu,[‡,#] Rahul Kancharla,[¶] Jiaoyan Li,[§] Seyed M. Hatamlee,[§] Gang Ren,[‡] Viktoriya Semeykina,[‖] Ahmed Hamed,[⊥] and Joshua J. Kane[¶]

†*Energy and Environment Science & Technology, Idaho National Laboratory, Idaho Falls, ID 83415, USA*
‡*The Molecular Foundry, Lawrence Berkeley National Laboratory, Berkeley, CA 94720, USA*
¶*Materials & Fuels Complex, Idaho National Laboratory, Idaho Falls, ID 83415, USA*
§*Department of Mechanical and Aerospace Engineering, University at Buffalo, Buffalo, NY 14260, USA*
‖*Department of Chemistry, The University of Utah, Salt Lake City, UT 84112, USA*
⊥*Energy and Environment Science & Technology, Idaho National Laboratory, Idaho Falls, ID 83415, USA*
#These authors contributed equally to this work.

E-mail: yidong.xia@inl.gov

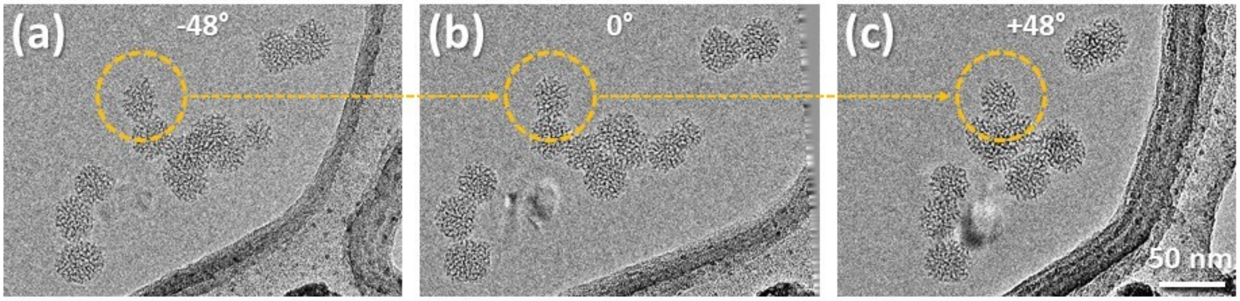

Figure S1. Three representative views of the cryo-ET tilt series of MSNs.

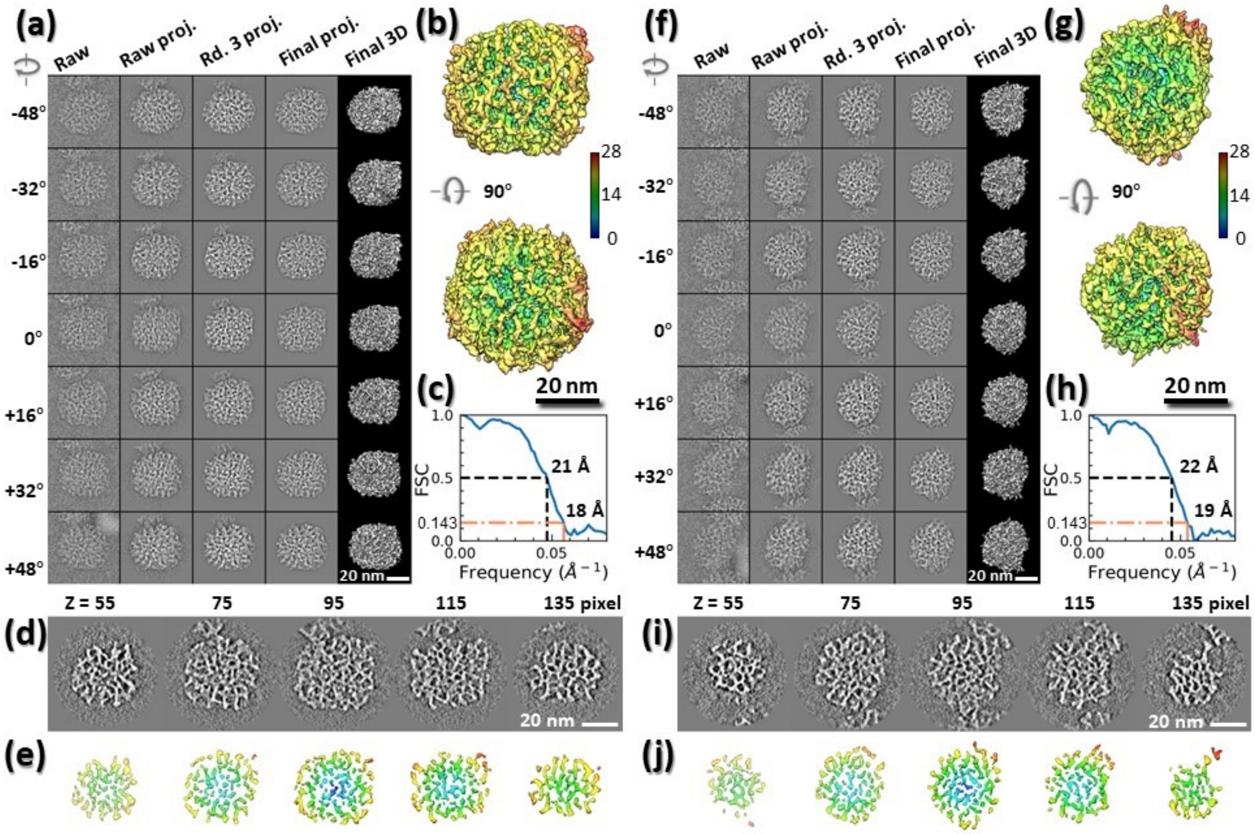

Figure S2. Cryo-ET 3D reconstruction of the 1st and 2nd particle of MSNs by IPET: **(a)** The process of 3D reconstruction of the 1st particle. Seven representative tilt images of the particle are gradually aligned via an iterative refinement. **(b)** Final 3D density map was colored by radius; the color bar indicates the distance from the geometry center of particle (unit: nm). (**c**) The FSC curve shows the resolution of the final 3D map was 18 Å at the criteria of 0.143. **(d)** The X-Y slices (thickness = 1.7 nm) of the spherical masked 3D map at different Z heights (Box size: X = Y = Z = 192 pixels; 1 pixel = 3.34 Å). **(e)** The X-Y slices (thickness = 1.7 nm) of the iso-surface rendered final 3D map at different Z heights. **(f-j**) The process of 3D reconstruction of the 2nd particle. The FSC curve shows the resolution of the final 3D map was 19 Å at the criteria of 0.143.

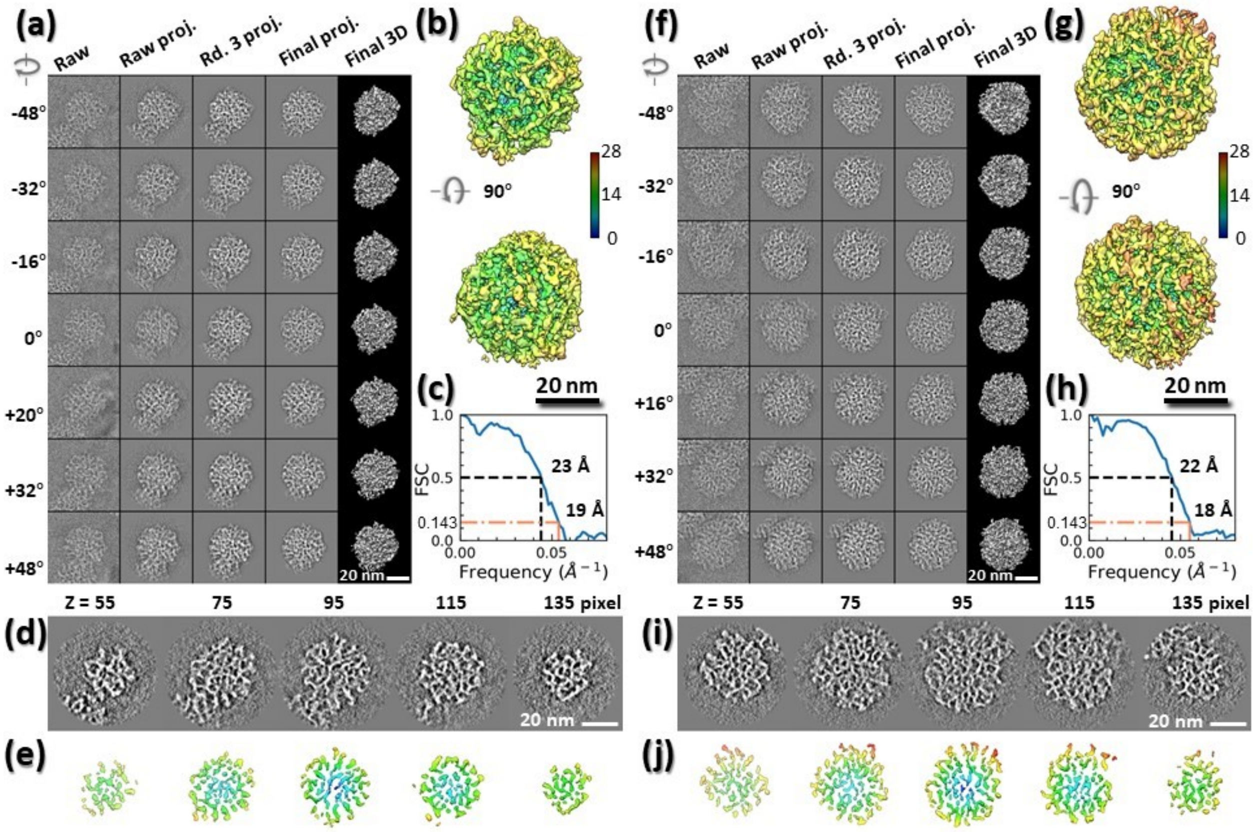

Figure S3. Cryo-ET 3D reconstruction of the 3rd and 4th particle of MSNs by IPET: **(a)** The process of 3D reconstruction of the 3rd particle. Seven representative tilt images of the particle are gradually aligned via an iterative refinement. **(b)** Final 3D density map was colored by radius; the color bar indicates the distance from the geometry center of particle (unit: nm). **(c)** The FSC curve shows the resolution of the final 3D map was 19 Å at the criteria of 0.143. **(d)** The X-Y slices (thickness = 1.7 nm) of the spherical masked 3D map at different Z heights (Box size: X = Y = Z = 192 pixels, 1 pixel = 3.34 Å). **(e)** The X-Y slices (thickness = 1.7 nm) of the iso-surface rendered final 3D map at different Z heights. **(f-j)** The process of 3D reconstruction of the 4th particle. The FSC curve shows the resolution of the final 3D map was 18 Å at the criteria of 0.143.

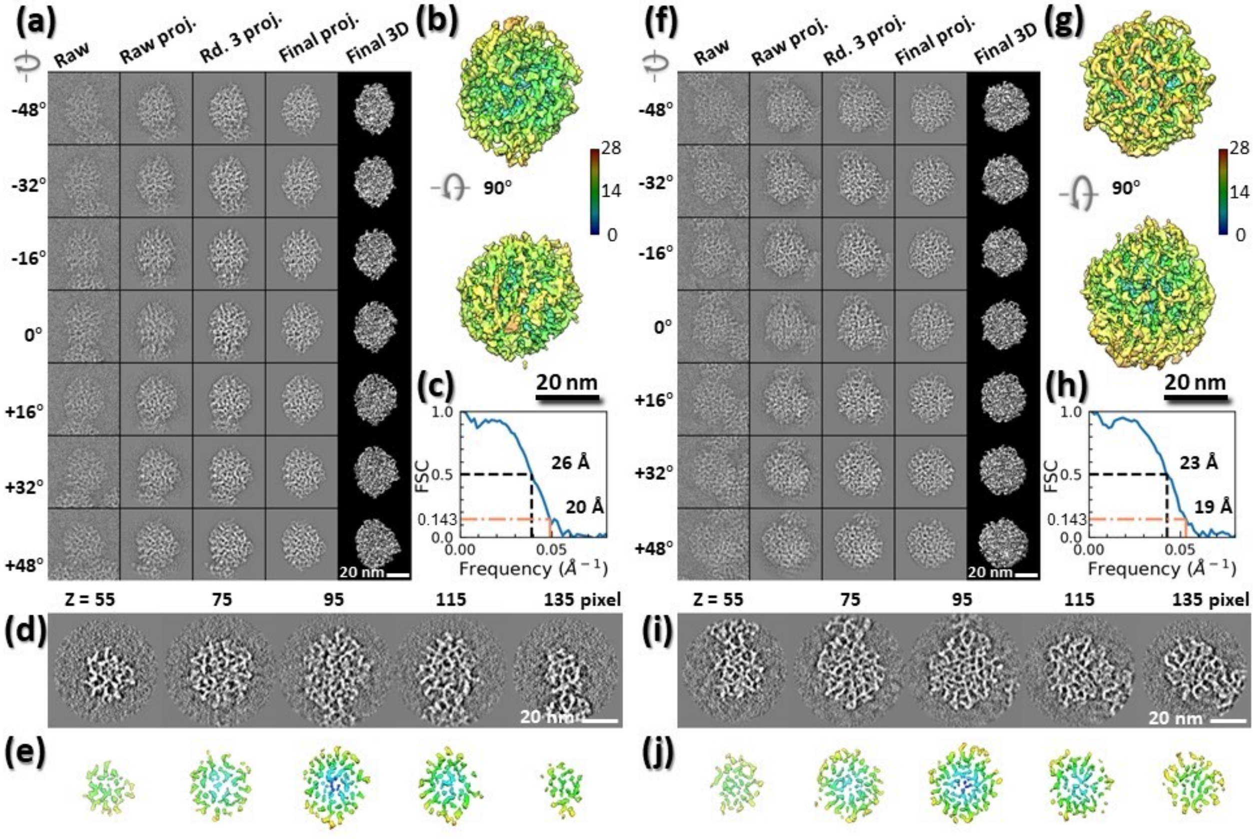

Figure S4. Cryo-ET 3D reconstruction of the 5th and 6th particle of MSNs by IPET**: (a)** The process of 3D reconstruction of the 5th particle. Seven representative tilt images of the particle are gradually aligned via an iterative refinement. **(b)** Final 3D density map was colored by radius; the color bar indicates the distance from the geometry center of particle (unit: nm). **(c)** The FSC curve shows the resolution of the final 3D map was 20 Å at the criteria of 0.143. **(d)** The X-Y slices (thickness = 1.7 nm) of the spherical masked 3D map at different Z heights (Box size: X = Y = Z = 192 pixels; 1 pixel = 3.34 Å). **(e)** The X-Y slices (thickness = 1.7 nm) of the iso-surface rendered final 3D map at different Z heights. **(f-j)** The process of 3D reconstruction of the 6th particle. The FSC curve shows the resolution of the final 3D map was 19 Å at the criteria of 0.143.

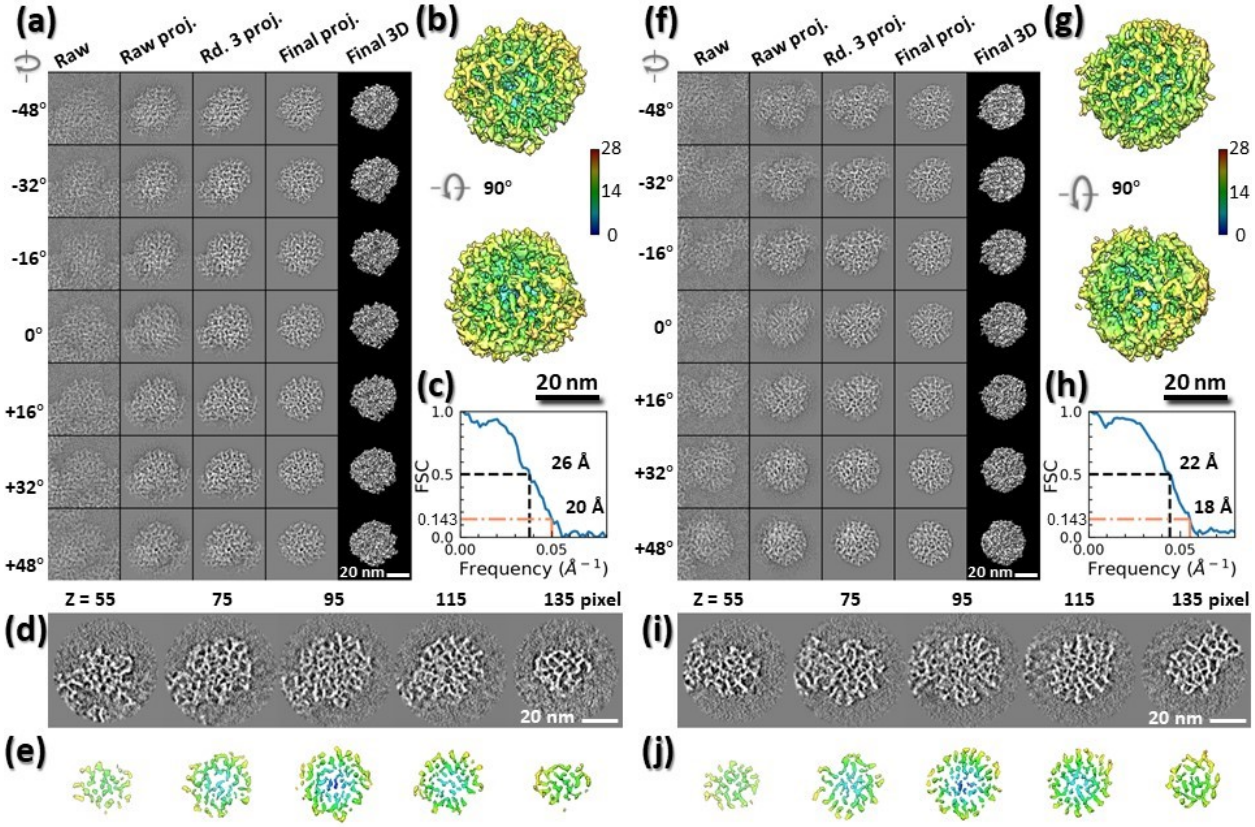

Figure S5. Cryo-ET 3D reconstruction of the 7th and 8th particle of MSNs by IPET: **(a)** The process of 3D reconstruction of the 7th particle. Seven representative tilt images of the particle are gradually aligned via an iterative refinement. **(b)** Final 3D density map was colored by radius; the color bar indicates the distance from the geometry center of particle (unit: nm). **(c)** The FSC curve shows the resolution of the final 3D map was 20 Å at the criteria of 0.143. **(d)** The X-Y slices (thickness = 1.7 nm) of the spherical masked 3D map at different Z heights (Box size: X = Y = Z = 192 pixels; 1 pixel = 3.34 Å). **(e)** The X-Y slices (thickness = 1.7 nm) of the iso-surface rendered final 3D map at different Z heights. **(f-j)** The process of 3D reconstruction of the 8th particle. The FSC curve shows the resolution of the final 3D map was 18 Å at the criteria of 0.143.

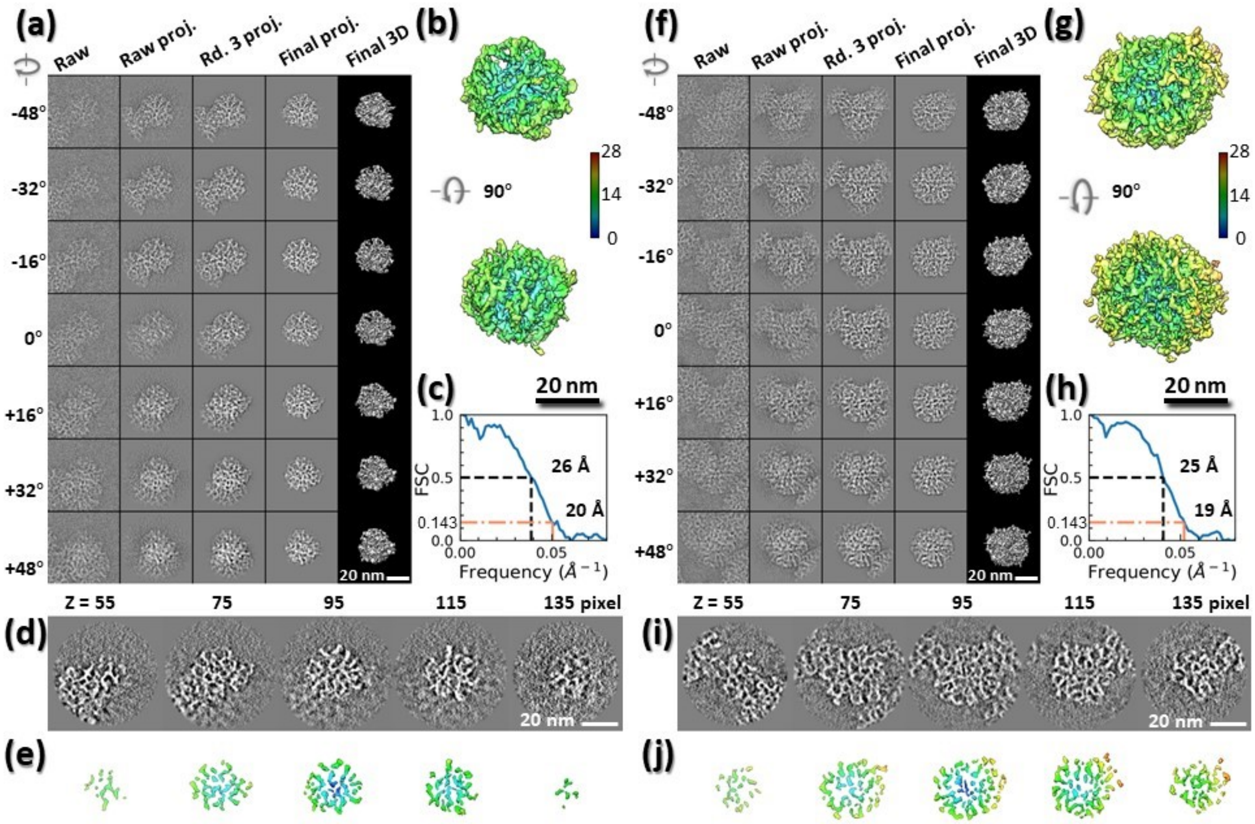

Figure S6. Cryo-ET 3D reconstruction of the 9th and 10th particle of MSNs by IPET: **(a)** The process of 3D reconstruction of the 9th particle. Seven representative tilt images of the particle are gradually aligned via an iterative refinement. **(b)** Final 3D density map was colored by radius; the color bar indicates the distance from the geometry center of particle (unit: nm). **(c)** The FSC curve shows the resolution of the final 3D map was 20 Å at the criteria of 0.143. **(d)** The X-Y slices (thickness = 1.7 nm) of the spherical masked 3D map at different Z heights (Box size: X = Y = Z = 192 pixels; 1 pixel = 3.34 Å). **(e)** The X-Y slices (thickness = 1.7 nm) of the iso-surface rendered final 3D map at different Z heights. **(f-j)** The process of 3D reconstruction of the 10th particle. The FSC curve shows the resolution of the final 3D map was 19 Å at the criteria of 0.143.
